# Hierarchical assembly of mixed 2D surfactants in polyHIPEs: tuning conductive networks through multi-scale structuring

**DOI:** 10.1039/d6ra01377e

**Published:** 2026-04-28

**Authors:** Deep Shikha Srivastava, Elizabeth E. B. Brown, Deepthi Varghese, Douglas H. Adamson

**Affiliations:** a Department of Chemistry, University of Connecticut Storrs CT 06269 USA Adamson@UConn.edu; b Polymer Program, Institute of Materials Science, University of Connecticut Storrs CT 06269 USA

## Abstract

Hierarchical assembly of two-dimensional (2D) surfactants within high internal phase emulsions (HIPEs) provides a direct route to multifunctional open-cell foams; however, the relationships between particle distribution, emulsion architecture, and macroscopic properties remain poorly understood. In this study, we investigate polyHIPE composites stabilized by dual 2D surfactants by systematically varying surfactant type, size, and mixing protocols. Specifically, we examine emulsions stabilized either by graphene sheets of distinct lateral sizes (1 µm and 10 µm) or combinations of graphene and boron nitride (BN) sheets. Additionally, we explore how surfactant mixing order, pre-mixed (combining surfactants before emulsification) *versus* post-mixed (combining separately emulsified components), influences emulsion stability and foam morphology. We observed cell-size variation in pre-mixed, 1 µm *vs.* 10 µm polyHIPES, with the size varying from 192 µm (for 1 µm control) to ∼1076 µm (for 10 µm control) and 280 µm for the pre-mixed samples. Pre-mixed systems demonstrated that 2D surfactants were well-mixed into the emulsion cell walls, whereas post-mixed emulsions retained distinct phase-separated shapes. The resulting polyHIPEs demonstrated promise in controlling electrical conductivity (0.31 S m^−1^ for post-mixed G-BN polyHIPEs *vs.* undetectable for pre-mixed G-BN polyHIPEs) enabling the creation of composites with tunable conductive properties. They also exhibited good viscosity and structural strength, making them suitable for use in 3D-printable material systems. This research provides new insights for designing functional composite materials using 2D surfactant combinations, thereby opening up new possibilities for next-generation emulsion-templated materials.

## Abstract 2

Assembly of 2D surfactants within high internal phase emulsions (HIPEs) provides a route to multifunctional open-cell foams; here, the relationships between particle distribution, emulsion architecture, and macroscopic properties are investigated.

## Graphical abstract

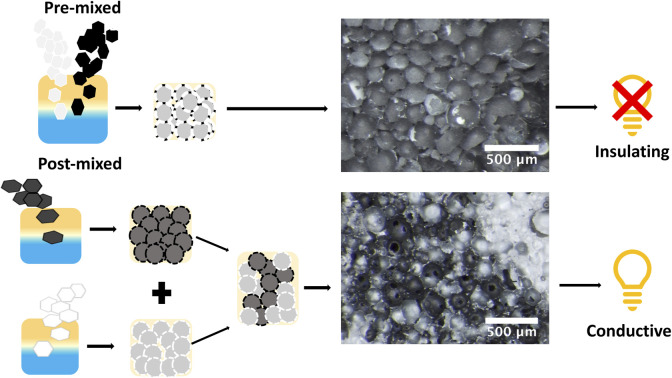

## Introduction

The geometric and thermodynamic differences between low- and high-aspect-ratio particles set 2D surfactant-stabilized systems apart from traditional particle-stabilized emulsions and present a facile route to composite production. Particle-stabilized emulsions were first reported in the literature in 1903 (ref. 1) and have been widely studied for applications in advanced material synthesis, biomedicine, food, and energy storage.^2–8^ Compared to small molecule surfactants, particle-stabilized emulsions are more stable,^2^ but when used as templating agents for high internal phase emulsion (HIPE) materials, they produce closed-cell foams rather than the open-cell foams desired for polymerized HIPE (polyHIPE) materials. High aspect ratio 2D materials such as graphene have been shown to combine the strength of particles with the open-cell morphology of surfactants to produce high-strength open-cell polyHIPEs with the added advantage of incorporating the conductive properties of the graphene.^9^ Emulsions templated by high aspect ratio materials (generally flat or platelike), including graphene and its analogue hexagonal boron nitride and molybdenum disulfide, are still a burgeoning and under-explored field.^10–12^

Of the two-dimensional stabilizers, graphene and its derivatives are among the most popular due to their high mechanical strength, electrical and thermal conductivity, and optical transparency.^13–16^ Though graphene is often functionalized with oxygen groups and subsequently reduced to aid in its dispersibility,^17^ emulsions and composite materials with pristine graphene have been prepared *via* the solvent interfacial trapping method (SITM).^18^ Pristine graphene's lack of dispersibility in either oil or water makes it an effective emulsion stabilizer: it readily exfoliates by spreading at high-energy aqueous/organic interfaces to lower the interfacial energy of the system.^10–12^ This principle is exploited to create polyHIPEs templated by the exfoliated graphene-stabilized emulsions. PolyHIPEs stabilized by 2D particles are especially noteworthy, as the composites are imbued with the properties of the particulate surfactant used. For example, graphene provides improved mechanical strength and electrical conductivity to a polymer matrix, and boron nitride imparts improved thermal conductivity and oxidative resistance.^19–22^

In addition to the properties gained by the presence of a single type of 2D filler, hybrid systems have also been demonstrated.^23^ The most common hybrid filler systems in emulsion-templated systems appear to be oppositely charged particles with low aspect ratios^24–26^ or materials such as graphene oxide decorated with metallic nanoparticles.^27,28^ Hybrid 2D fillers are far more common in other systems. Coleman *et al.*^29^ reported synthesizing free-standing films that combine either boron nitride, MoS_2_, or WS_2_ with graphene, and several others have combined graphene and boron nitride to enhance the composite's thermal and mechanical properties.^20,30–32^

Despite the promising results with free-standing films, combining 2D materials with different sizes or chemistries into polyHIPE composites has not been reported. The work presented here explores 2D surfactant-stabilized systems incorporating particles of different sizes and chemistries at three distinct length scales: nanometer-scale films of overlapping 2D sheets, micron-scale polyHIPE cells, and millimeter-sized features formed by a 3D printing approach. We find that we can control the cell size and size distribution, as well as the composites' electrical conductivity, by controlling the particle distribution and we investigate the mechanism behind this control. The impact of the composite's morphology on its properties is studied, as well as the integrity of the graphene percolating network when incorporated into a system with boron nitride. Finally, we demonstrate a technique for patterning a composite using a 3D printing approach, enabling the creation of composites with complex designs and tunable properties.

## Experimental

### Materials

Graphite: natural 1 µm flake size (2299, Asbury Carbons), natural 10 µm flake size (Micro 890, Asbury Carbons). Boron nitride: 2 µm platelet (IDL 600, St. Gobain). Styrene (Reagent-plus, Sigma-Aldrich), divinylbenzene (DVB, technical grade, 80%, Aldrich), and 2,2′-azobis(2-methylproprionitrile) (AIBN, 98%, Aldrich) were all used as received.

### Synthesis

#### Pre-mixed particle-stabilized composites

All emulsions were prepared with a graphite or h-BN loading of 4.4 mg mL^−1^ relative to the sum of the aqueous and oil phases. The water-to-monomer ratio was maintained at 7/3, with more details available in the SI. A typical emulsion was prepared in an Erlenmeyer flask by combining 0.88 g of graphite, boron nitride, or a combination of the two, 140 mL of deionized water, 60 mL of styrene, 14 mL of DVB, and 0.18 g of AIBN. The reagents were stirred on a magnetic stir plate using a stir bar for ∼1 minute, then poured into a 200 mL jar and shaken in a bubble tea shaker (Happybuy Milk Tea) for 1 minute. The jars were then placed in a 60 °C oven for 12 hours, after which they were opened and the composites left in the oven for an additional 2–3 days to dry.

#### Post-mixed particle-stabilized composites

**Scheme 1:**
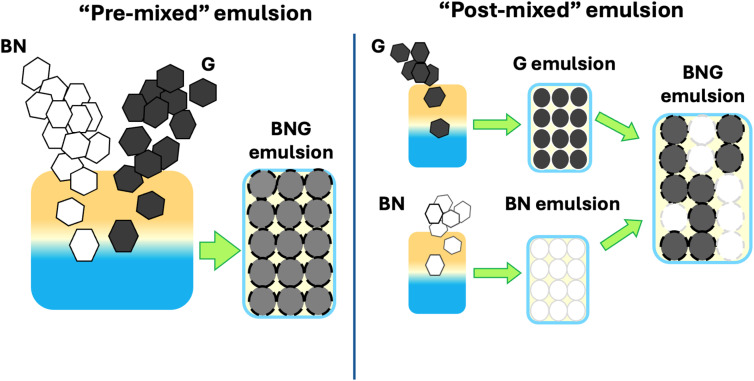
An illustration of the “pre” and “post” mixing methods of making particle-stabilized emulsions with more than one type of surfactant. The crucial difference between the two is that in the post-mixing method, each species of particle is used to stabilize two separate emulsions, then these emulsions are combined into one container. In the pre-mixing method, both species are combined at the start and form one singular emulsion.

The boron nitride and/or graphene emulsions were prepared as described in the previous section. Then, immediately after each emulsion was shaken, they were simultaneously poured into a 200 mL jar. The jar was then capped, gently rotated, and turned several times by hand to ensure the mixing of the separate emulsions. The polymerization procedure and drying were the same as for the pre-mixed composites. More detailed information about the synthesis can be found in Scheme 1 and the SI (Tables S1–S4).

#### Graphene and boron nitride emulsions for 3D printing

The emulsions made to create the 3D-printed graphene/boron nitride composites were similar to those listed above. The graphene emulsion was made using 1 µm graphite at 8.8 mg mL^−1^, and the boron nitride emulsion was made using 2 µm boron nitride also at 8.8 mg mL; full details are described in the SI. The increased particle loading resulted in more viscous emulsions that better retained their shape when laid out for printing.

#### Fabrication of 3D printed composites

Three composites were made using a modified form of 3D printing: two in which one emulsion was pipetted on top of another (composites A and B; see Fig. 6) and a third produced using a layer-by-layer assembly (composite C). For composites A and B, ∼30 mL of graphene or boron nitride emulsion was poured into a 100 mL jar. The boron nitride chevron was created by pipetting a boron nitride emulsion with an 8.8 mg mL^−1^ concentration into a plastic pipette, then squeezing this emulsion onto the graphene emulsion, much like a piping bag is used to decorate a cake. Similarly, the graphene dot pattern was made by pipetting an 8.8 mg mL^−1^ graphene emulsion and then carefully depositing it on top of the boron nitride emulsion. The layer-by-layer composite C was created using a similar pipetting technique, and layers of boron nitride and graphene emulsions were carefully deposited to form the yin-yang pattern.

### Characterization

#### Scanning electron microscopy

Samples were mounted onto aluminum stubs affixed with carbon tape, then sputter-coated with an Au/Pd. Composite samples were imaged using an FEI Nova NanoSEM 450 with a spot size of 2.0 nm and an accelerating voltage of 5.0 kV.

#### Electrical conductivity

Samples from the bottom most portion of the composites were cut into rectangular cuboids with dimensions of approximately 1 cm × 1 cm × 3 cm. Samples were taken from the bottom portion of each composite to ensure consistent sampling. The emulsion droplets are closely packed throughout the bulk of the composite, but the top surface sometimes contains a layer of polymer resulting from emulsion drainage. Sampling from the bottom avoids this region and provides constant material for comparison. Colloidal silver paste (Ted Pella) was applied to the 1 cm^2^ ends of the sample and then allowed to dry overnight. A piece of copper tape was applied to each silver-pasted end, and alligator clips were applied to each piece of tape. The clips were connected to a Keithley 2420 sourcemeter, and conductivity was measured over a voltage range of 0.001 to 0.1 V. Current was passed along the 3 cm axis of the sample. Since the polyHIPE structure is isotropic, significant anisotropy in conductivity is not expected in the samples without patterning. Resistance was calculated from the slope of the resulting voltage-*versus*-current plot. Representative *I*–*V* curves for selected compositions are provided in SI Fig. S1, confirming linear ohmic behavior over the measurement range (0.001–0.1 V).

#### Mechanical testing

For sample preparation, depending on the length of the composites, samples were cut into one or two 1 cm slices or 1 cm × 1 cm × 1 cm cubes. The compression testing was performed using a 50 kN load cell, with a maximum strain of up to 70%, and a strain rate of 1.3 mm min^−1^, on an Instron 5869. Samples were tested in triplicate. A representative stress/strain curve is shown in SI Fig. S2.

#### Optical microscopy

Optical images were captured using a Leica M-125 stereomicroscope equipped with a Nikon D3500 camera. A series of images was taken for each composite sample and then stacked together using Helicon 7.0 Focus Stacking software.

#### Cell size analysis

Cell sizes were measured *via* ImageJ software. Around 175–200 cells were measured for each composite. The areas calculated by ImageJ were summed to obtain the total area of the image occupied by cells within a particular “size bin”. Cell sizes were measured from 2D cross-sectional images, and the area fraction of each size bin was used as a proxy for volume fraction. This 2D-to-3D approximation can introduce stereological bias, particularly for non-spherical cells or broad size distributions, because larger cells are preferentially intersected by a random cross-section. The reported distributions should therefore be interpreted as semi-quantitative.

## Results and discussion

### Graphene composites with mixed sheet sizes

**Fig. 1:**
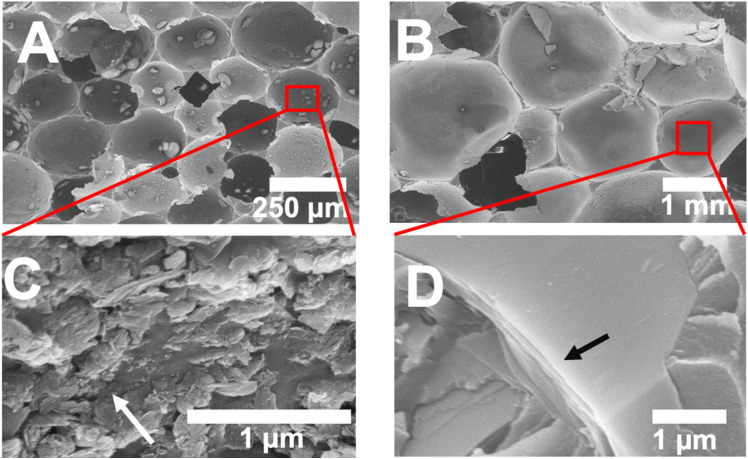
SEM images of graphene/polystyrene composites. (A) Overview of a composite made using 1 µm graphite, with a box zooming in (approximately) to show (C) the graphene sheets lining the interior of (A), with an arrow pointing to a small graphite cluster; (B) a composite made using 10 µm graphite and a box zooming in (approximately) to show (D) multiple stacked sheets of 10 µm sheets lining the cell interior of (B). Note that the scale bars in (A) and (B) are different.

We have previously shown that the size of emulsion spheres and the subsequent cells of the polyHIPEs are governed by the lateral dimensions of the graphene, as there is a limit to how much each graphene sheet can bend to conform to the curvature of the water droplets.^12^ However, it is not clear what effect mixed sheet sizes would have on the morphology of the composite. To investigate this, we first synthesized two control samples: one using natural flake graphite with a flake size centered around 1 µm and another using natural flake graphite with a flake size centered around 10 µm. Fig. 1 shows SEM images of those composites. The cells formed by 1 µm graphite in Fig. 1A averaged between 150–300 µm in diameter and maintained a spherical morphology. In contrast, the 10 µm-based composite in Fig. 1B had distorted cells nearly an order of magnitude larger in diameter than the 1 µm control. The polyhedral cell shape occurred due to the lower Laplace pressure of the larger spheres. As the droplet radius increased, the pressure within the cell diminished until it was less than the pressure exerted by the surrounding liquid, resulting in droplet deformation.^33^ The cells in these materials also tended to remain closed, in contrast to the windows between cells that appeared in the 1 µm composite.

The difference in the size of the spheres is not solely a result of graphene's stiffness, however. We have shown previously that there is a direct correlation between the amount of graphite added and the size of the water droplets, down to a minimum size that is controlled by sheet stiffness.^18^ This is simply a result of smaller spheres having a larger interfacial area for the same encapsulated volume compared to larger spheres. Additionally, since the larger graphite flakes exfoliate more slowly than the smaller flakes,^34^ there is less graphene, and thus less interfacial area can be stabilized. This is the case with small molecule surfactants as well as particle-stabilized emulsions, which become smaller and rounder with increased solid concentration.

Fig. 1C and D show higher magnification images of the 1 µm and 10 µm graphene-based composites, respectively. The cell surface shown in Fig. 1C shows graphite embedded and protruding from the polymer. Atomically thin graphene is not observed at this magnification, due to both its dimensions and to being buried in the polymer. Computational modeling has shown that the graphene lies on the oil side of the oil/water interface,^35^ and is thus encased in the polymer. Larger graphite flakes that did not exfoliate but were pinned at the interface during polymerization are observed spanning the oil/water interface. Once an overlapping film of graphene sheets stabilizes the oil/water interface, the driving force for exfoliation no longer exists, so excess graphite remains. The presence of exfoliated graphene sheets has been confirmed previously by TEM images of microtomed samples.^36^ For simplicity, we refer to the interface as being stabilized by graphene, although some unexfoliated graphite is also present.

Compared to the 1 µm graphite composite, the 10 µm graphite material shows large graphite flakes that do not appear to be embedded in the polymer and appear parallel to the interface rather than perpendicular to it. Kinetic studies have shown that larger sheets exfoliate less readily than smaller sheets,^34^ and while this may play a role, it is more likely that the size of the graphite flake relative to the width of the water/oil interface explains the difference in graphite orientation. Our previous free-energy calculations show that the energetic penalty associated with graphene moving either into the water phase or the oil phase increases with increasing distance from the interface. Thus, an orientation perpendicular to the interface is considerably less likely for a 10 µm flake than a 1 µm flake, resulting in the larger flakes tending to be oriented parallel to the interface.

**Fig. 2:**
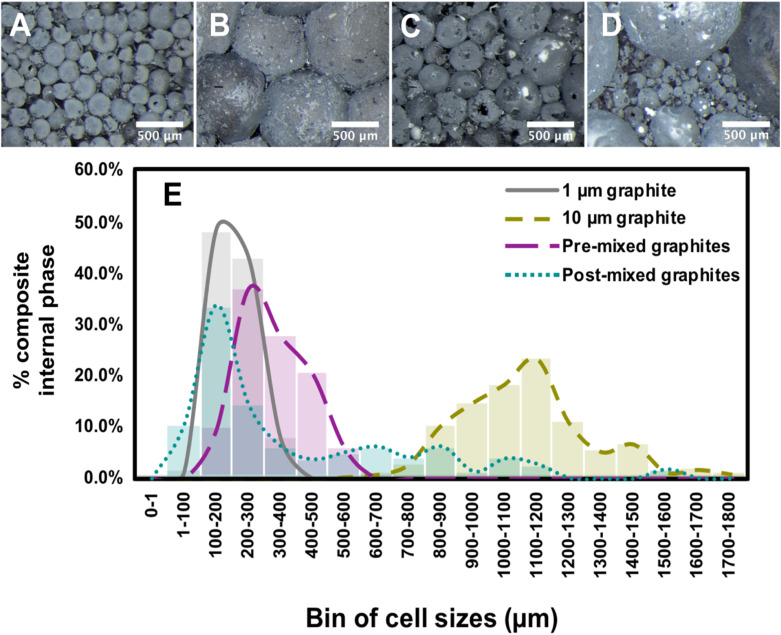
Optical microscopy images of composites made with (A) 100% 1 µm graphite; (B) 100% 10 µm graphite; (C) a 1 : 1 mixture of 1 µm and 10 µm graphite with cell sizes between those of (A) and (B); (D) a 1 : 1 combination of a 1 µm and a 10 µm emulsion, with two unique populations of cell sizes; (E) chart depicting the percentage of composite area comprised of each bin of sphere sizes in polymer graphene composites. See SI Fig. S3 for larger versions of the images.

Although the role of sheet size in determining the diameter of the droplets has been addressed previously,^10^ the effect of mixing two sheet sizes on the droplet diameter has not. We prepared a series of composites incorporating both 1 µm and 10 µm sheets using both a “pre-mixed” and a “post-mixed” method: in the pre-mixed approach, two graphite flake sizes were added to one flask and emulsified, while in the “post-mixed” method two separate emulsions were made using each flake size separately, followed by combining these emulsions before polymerization. Fig. 2A and B show polyHIPEs made with only one flake size, and these served as controls. Fig. 2C shows the material prepared by pre-mixing, while Fig. 2D shows the material made with the same components as in Fig. 2C but using the post-mix approach. What is immediately apparent is that in the sample made by pre-mixing, the distribution of cell sizes is much narrower than in the post-mix material, and the cell size is roughly the average of the sizes from the controls made with each graphite size. When two emulsions were combined post-emulsifying, however, the polyHIPEs contained two distinct sphere sizes, with the size of the large droplets being somewhat smaller than when just 10 µm graphite was used. We hypothesize that the 10 µm-based emulsion droplets, which would otherwise have had insufficient graphene coverage to resist coalescence, were stabilized by the smaller droplets surrounding them. This is supported by numerous holes in these cells, which typically only form when there is little or no polymer between two adjacent droplets^37^ and portions of the graphene cell wall rupture upon drying.^38^

Using image analysis, the population of cell sizes in the different polyHIPEs was quantified and is shown in Fig. 2E. Histograms of each polyHIPEs separately are shown in SI Fig. S4. The percentage of the total internal foam volume by cell size is plotted for the four polyHIPE materials shown in Fig. 2A–D. In this analysis, we set the volume percentage as the area percentage of each sphere in each two-dimensional image. The data is presented in terms of volume rather than number to better illustrate the physical make-up of the polyHIPEs.

For the 1 µm graphite polyHIPEs, the cell size was fairly unimodal, with 43% and 48% of the volume consisting of 100–200 and 200–300 µm spheres, respectively. No cells measured larger than 500 µm, whereas all cells in the 10 µm graphite sample were over 600 µm in diameter, with many exceeding 1 mm. In the pre-mixed material, the cell-size dispersity broadened, with cell sizes ranging between those of the purely 1 µm and purely 10 µm graphite-based materials. The average cell size of the pre-mixed material was ∼280 µm, compared to ∼192 µm for the 1 µm and ∼1076 µm for the 10 µm polyHIPEs. The cell sizes in the pre-mixed sample may skew smaller than the average of the two controls because the 1 µm graphite exfoliates more quickly than the 10 µm material, meaning that 1 µm graphene is more abundant and plays an outsized role in stabilizing dispersed water droplets.^34^ While the SEM images indicate that both sheet sizes are incorporated into the emulsion spheres, a greater percentage of the stabilizing graphene is likely of the smaller size.

The situation with the post-mixed emulsion was quite different, with cell sizes exhibiting a polymodal distribution. Approximately 32% of the composite contained larger spheres, reflecting the increased graphene-stabilized interface of polyHIPEs made with smaller graphite flakes. Overall, the larger spheres in the post-mixed material were smaller when compared with those in the control 10 µm sample. It is likely that in this scenario the presence of adjoining 1 µm graphene-based droplets stabilizes neighboring 10 µm-based spheres before those spheres coalesce. In essence, the smaller spheres act as additional exfoliated graphite to provide stability.

**Fig. 3:**
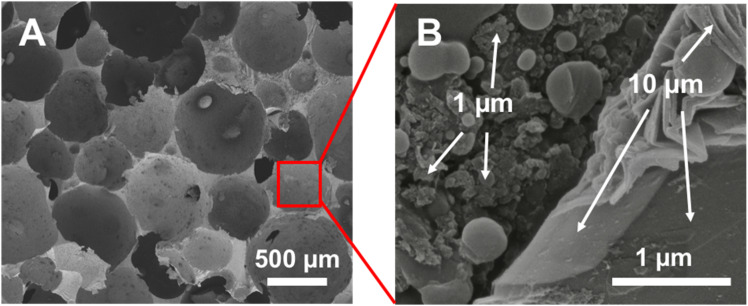
SEM of an emulsion-templated pre-mixed composite made using a 1 : 1 ratio of 1 µm and 10 µm sheet sizes. (A) Overview of composite; (B) in-depth magnification of cell interior to show both 10 µm sheets and 1 µm sheets. The small globules are polystyrene beads that precipitated from the aqueous phase during polymerization.

To verify that the individual spheres in the pre-mixed composites contained a mixture of the two sheet sizes, SEM images (Fig. 3) were obtained. These images verify that 1 and 10 µm graphene sheets are incorporated into the cell walls and stabilize the emulsion droplets. Fig. 3A provides an overview of the composite and the cell sizes, while Fig. 3B presents a high-magnification image of the interior of one of these cells, revealing two distinct sizes of graphite sheets. Several small beads or misshapen blobs are also visible in the picture; these are polystyrene from styrene that dissolved in the water during the emulsion phase and then precipitated out of the solution during polymerization.

**Fig. 4:**
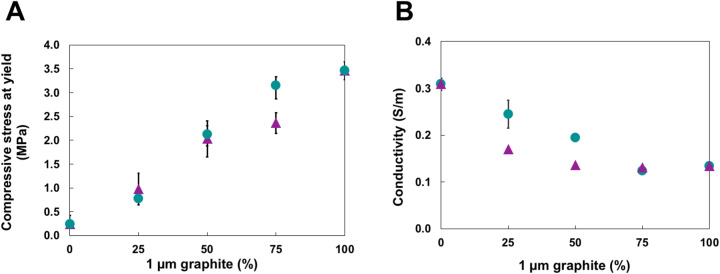
Graphs depicting (A) the compressive stresses at yield and (B) electrical conductivities of composites made using varying concentrations of 1 µm graphite relative to 10 µm graphite. Pre-mixed samples are depicted by purple triangles, and post-mixed samples are shown as teal circles. Error bars represent the standard deviation of *n* = 3 independent samples per composition.

The extent of graphene sheet mixing within the spheres is also reflected in the composite's properties, such as compressive strength and electrical conductivity. Fig. 4A shows a generally increasing trend of compressive strength with a higher percentage of 1 µm graphite, which agrees with previous comparisons between 1 and 10 µm graphite-based composites.^18^ A more significant portion of 10 µm graphite in the pre-mixed samples creates larger, less robust cells that fracture more easily, as the reduced exfoliation of the 10 µm graphite results in less material incorporated into the cell wall. In the post-mixed samples, the more fragile 10 µm-based cells fracture before the sturdier 1 µm-based cells, leading to lower compressive strengths as the fraction of 1 µm graphite is reduced. The most striking difference is between the pre- and post-mixed samples at a concentration of 75% 1 µm graphite: the post-mixed composite is notably stronger, with an average compressive strength of 3.15 MPa compared to 2.36 MPa for the pre-mixed sample. It appears that at or around this ratio of graphite, sufficient small spheres surround larger spheres to reinforce deformation. At the same time, the slightly increased cell size and reduced graphene content of the pre-mixed foams results in a reduction of mechanical properties. An alternative version of this graph is presented in SI Fig. S5, where the compressive stresses at yield are normalized by foam density to ensure that abnormally high or low composite densities do not skew the data. The trend is very similar to the one shown in Fig. 4A, suggesting that the incorporation of graphene in the cell walls, rather than the increased density resulting from added graphite, differentiates the mechanical properties between the pre- and post-mixed composites.

In the post-mixed composites, separately emulsified graphene and h-BN droplets are combined *via* gentle inversion prior to polymerization. Because the continuous oil phase is common to both emulsions, it forms a single, continuous matrix upon mixing. The free-radical copolymerization of the styrene and DVB oil phase creates a covalently crosslinked polymer matrix around each sphere, regardless of which particle type stabilizes the sphere. Thus, the mechanical integrity of the post-mixed composite does not rely on adhesion between dissimilar particles. Instead, it is a function of the crosslinked polystyrene/DVB network and the cell wall strength.

Fig. 4B demonstrates the influence of graphene sheet distribution on electrical conductivity. The conductivity was measured using a two-point probe, with silver epoxy applied to reduce contact resistance. Larger graphene sheets facilitate greater conductivity, requiring less charge hopping from sheet to sheet.^16^ We found that composites made using solely 10 µm graphite had an average conductivity of 0.31 S m^−1^, compared to 0.13 S m^−1^ for the 1 µm composites. Notably, we found that the sample's conductivity was a function of the mixing approach. In the pre-mixed material, conductivity dropped significantly after adding only 25% 1 µm graphite to the 10 µm emulsion compared to a much smaller decrease when two emulsions were combined post-mixing. In the pre-mixed sample, the two graphene sizes are more uniformly mixed and are, therefore, evenly incorporated into the percolating network. In post-mixed composites, conduction pathways that are nearly entirely composed of large sheets still exist, and thus, the observed conductivity does not decrease as dramatically. Table S5 of the SI includes the electrical conductivity values for 1 µm and 10 µm graphene composites featuring different percentages of each type of graphene. Additionally, the cell size data from Fig. 2 suggests that 1 µm graphene is preferentially incorporated into the composite compared to 10 µm graphene in the pre-mixed materials.

### Graphene/boron nitride composites

**Fig. 5:**
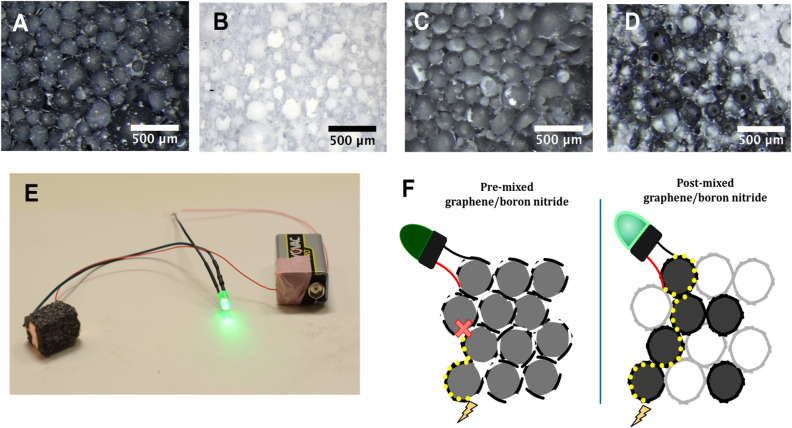
Optical microscopy images depicting boron nitride/graphene composites. (A) A composite made only with graphene; (B) A composite made only with boron nitride; (C) “pre-mixed” composite in which the boron nitride and graphene were combined prior to making an emulsion. (D) “Post-mixed” composite in which a boron nitride and graphite emulsion were each prepared separately, then combined together and polymerized. (E) LED illuminated by a circuit containing a piece of “post-mixed” boron nitride/graphene composite. (F) Cartoon illustration of current traveling along both a pre- and post-mixed boron nitride/graphene composite.

In addition to the particles differing in size, we used hexagonal boron nitride with graphene to study the effect of systems that varied in chemical composition. Because graphene emulsions are black and boron nitride emulsions are white, the stark visual contrast between the two quickly showed if the emulsions maintained discrete spheres when combined. Using a 1 : 1 ratio of boron nitride and graphene and employing the “pre-mixed” and “post-mixed” methods detailed in the Experimental section, we compared the black graphene and white boron nitride composites in Fig. 5A and B with the pre-mixed composite in Fig. 5C. We found the mixed composite appeared uniformly gray. The post-mixed composite, however, has black and white cells scattered throughout, indicating that the spheres of the two emulsions remained discrete when combined (Fig. 5D). The gray appearance of the pre-mixed composite is due to the simultaneous exfoliation of boron nitride and graphene, with a mixture of the two incorporated into each sphere. Conductivity measurements further support this, as the post-mixed sample was electrically conductive (Fig. 5E), whereas the pre-mixed sample was not. This indicates that when graphene and boron nitride are mixed before forming an emulsion, both undergo exfoliation at the solvent interface, resulting in an intimately mixed graphene/h-BN film at the nanoscale that prevents the formation of a conductive graphene network. When the two were combined at a larger length scale as separate emulsions after each had exfoliated separately, the graphene spheres formed a percolating network despite the presence of boron nitride spheres, as is indicated in Fig. 5F.

The uniform gray appearance of the pre-mixed composite (Fig. 5C), combined with its lack of electrical conductivity, is consistent with the simultaneous exfoliation and mixing of graphene and h-BN sheets at the oil–water interface during emulsification, since if this were the case, the electrically insulating h-BN sheets would interrupt the graphene–graphene contacts needed for electrical percolation. In contrast, the post-mixed composite retained distinct black and white regions (Fig. 5D) and remained electrically conductive (0.31 S m^−1^), indicating that the separately formed emulsion droplets preserved their original particle identity when combined. While these observations are consistent with nanoscale mixing, the proposed interfacial mixing mechanism is a plausible inference rather than a directly verified conclusion.

**Fig. 6:**
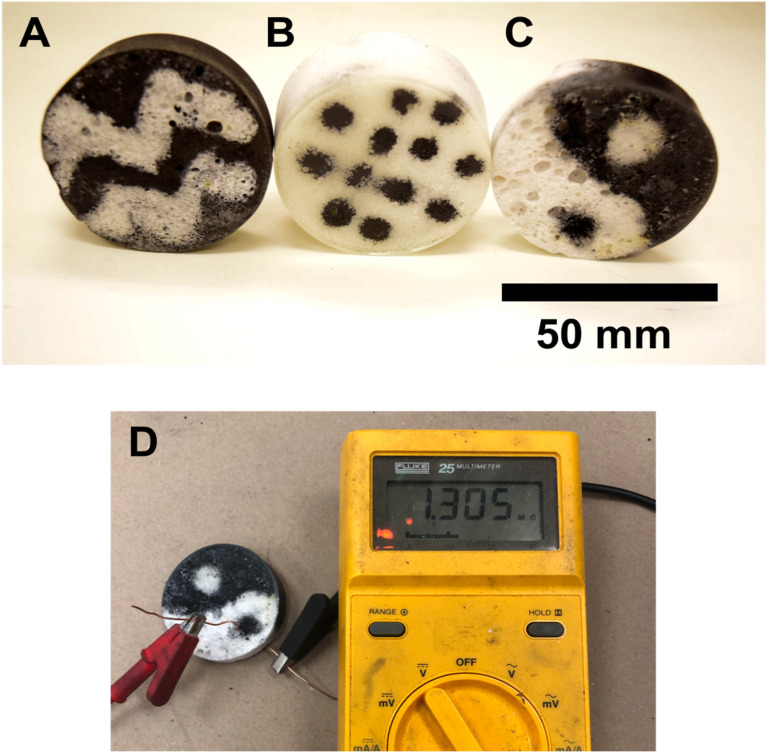
Composites made using 3-D printed emulsions. (A) Boron nitride printed on top of graphene. (B) Graphene printed on top of boron nitrides; (C) graphene and boron nitride assembled in a layer-by-layer technique; (D) electrical conductivity in the graphene “eye” of a graphene and boron nitride layer-by-layer composite.

Particle-based emulsions are exceptionally stable due to the high energy required to displace a particle from the liquid–liquid interface.^2^ The strong adhesion of the sheets to the emulsion spheres caused both the graphene and boron nitride emulsions to remain intact even with light agitation, such as being poured into the same vessel. This suggested that it might be possible to place spheres composed of different 2D particles into another emulsion prior to polymerization. Combined with the visual contrast in the boron nitride/graphene emulsions, we demonstrated rudimentary liquid–liquid 3D printing using our emulsions. The concept of 3D printing with liquids has been explored by Russell *et al.* using anionic nanoclay^39^ and cellulose nanocrystal.^40^ The process is straightforward; one can draw basic geometries such as dots, circles, and lines using a plastic pipette to add one emulsion to another or even form a layer-by-layer structure, as shown in Fig. 6. Chevron patterns of boron nitride were drawn in a graphene emulsion, and a pattern of graphene dots was added to a boron nitride emulsion. Although the spheres remain unbroken, some “bleeding” can occur if excess monomer floats to the top of the emulsion while the spheres settle, giving the printed spheres more freedom to move around. This bleeding can be minimized by removing extra monomer before patterning the emulsion.

This technique was also used to create a layer-by-layer assembly of boron nitride and graphene emulsions, as shown in the yin-yang composite in Fig. 6C. Using a plastic pipette, emulsion layers were drawn in the yin-yang pattern and built up to create a three-dimensional structure. As opposed to the composites in Fig. 6A and B, this pattern persists through the depth of the structure instead of existing only on the surface. This is far from the only structure that can be fabricated using this technique. Layers of boron nitride and graphene can be alternated to form “sandwich” structures, and finer pipette tips allow for more detailed work and the design of more complex patterns.

The layer-by-layer assembly ensures that the graphene remains conductive. We measured the electrical conductivity of the eye part of the yin-yang symbol, which not only reinforces how the emulsions retain their unique identities but also opens the pathway for printing 3D wires and more elaborate 3D structures with deliberately designed conductive pathways.

## Conclusion

This study shows how carefully controlling the spatial distribution of two-dimensional surfactants across three different length scales nanometers (interfacial films, either mixed or segregated), microns (emulsion droplets before and after mixing), and millimeters (liquid in liquid printing) can be a practical way to tailor the structure and properties of polyHIPE composites. Changing the graphene lateral size from 1 µm to 10 µm resulted in the cell diameter varying by a factor of ten or more (about 190 µm to 1100 µm), and the compressive strength increased from 1.1 MPa to 3.2 MPa when small sheets were placed around larger ones. Different chemistries further expanded the potential functionalities: intimate graphene-h-BN films were able to significantly reduce electrical conductivity, while segregation at micron- or millimeter-scales maintained conductivity up to 0.31 S m^−1^. The technique of patterning conductive networks within insulating matrices through pipette-based layer deposition provides a simple yet effective method for creating patterned electrodes, porous current collectors, or thermal interfaces. Overall, the hierarchical placement of 2D surfactants transforms particle-stabilized emulsions into a versatile platform, where mechanical strength, pore structure, and electrical properties can be optimized through intentional design.

## Conflicts of interest

There are no conflicts to declare.

## Supplementary Material

RA-016-D6RA01377E-s001

## Data Availability

The original contributions presented in this study are included in the article/supplementary information (SI). Further inquiries can be directed to the corresponding author. Supplementary information is available. See DOI: https://doi.org/10.1039/d6ra01377e.

## References

[cit1] Ramsay W. (1903). Separation of Solids in the Surface-Layers of Solutions and “suspensions” (Observations on Surface-Membranes, Bubbles, Emulsions, and Mechanical Coagulation)-Preliminary Account. Proc. R. Soc. London.

[cit2] Chevalier Y., Bolzinger M. A. (2013). Emulsions Stabilized with Solid Nanoparticles: Pickering Emulsions. Colloids Surf., A.

[cit3] Yang Y., Fang Z., Chen X., Zhang W., Xie Y., Chen Y., Liu Z., Yuan W. (2017). An Overview of Pickering Emulsions: Solid-Particle Materials, Classification, Morphology, and Applications. Front. Pharmacol.

[cit4] Carranza A., Pérez-García M. G., Song K., Jeha G. M., Diao Z., Jin R., Bogdanchikova N., Soltero A. F., Terrones M., Wu Q., Pojman J. A., Mota-Morales J. D. (2016). Deep-Eutectic Solvents as MWCNT Delivery Vehicles in the Synthesis of Functional Poly(HIPE) Nanocomposites for Applications as Selective Sorbents. ACS Appl. Mater. Interfaces.

[cit5] Zhang T., Sanguramath R. A., Israel S., Silverstein M. S. (2019). Emulsion Templating: Porous Polymers and Beyond. Macromolecules.

[cit6] Zhao Q., He F., Zhang Q., Fan J., He R., Zhang K., Yan H., Yang W. (2019). Microencapsulated Phase Change Materials Based on Graphene Pickering Emulsion for Light-to-Thermal Energy Conversion and Management. Sol. Energy Mater. Sol. Cells.

[cit7] Mert E. H., Yildirim H., Üzümcü A. T., Kavas H. (2013). Synthesis and Characterization of Magnetic polyHIPEs with Humic Acid Surface Modified Magnetic Iron Oxide Nanoparticles. React. Funct. Polym..

[cit8] Seeharaj P., Thasirisap E., Tridech C., Jindasuwan S. (2019). Magnetic Polyhipe Composites with Activated Carbon and Iron Oxide Nanoparticles. IEEE Trans. Magn..

[cit9] Brown E. E. B., Woltornist S. J., Adamson D. H. (2020). PolyHIPE Foams from Pristine Graphene: Strong, Porous, and Electrically Conductive Materials Templated by a 2D Surfactant. J. Colloid Interface Sci..

[cit10] Woltornist S. J., Oyer A. J., Carrillo J. M. Y., Dobrynin A. V., Adamson D. H. (2013). Conductive Thin Films of Pristine Graphene by Solvent Interface Trapping. ACS Nano.

[cit11] Creighton M. A., Ohata Y., Miyawaki J., Bose A., Hurt R. H. (2014). Two-Dimensional Materials as Emulsion Stabilizers: Interfacial Thermodynamics and Molecular Barrier Properties. Langmuir.

[cit12] Woltornist S. J., Carrillo J. M. Y., Xu T. O., Dobrynin A. V., Adamson D. H. (2015). Polymer/Pristine Graphene Based Composites: From Emulsions to Strong, Electrically Conducting Foams. Macromolecules.

[cit13] Balandin A. A., Ghosh S., Bao W., Calizo I. (2008). Superior Thermal Conductivity of Single-Layer Graphene. Nano Lett..

[cit14] Lee C., Wei X., Kysar J. W., Hone J. (2008). Mechanical Properties of Monolayer Graphene. Science.

[cit15] Pop E., Varshney V., Roy A. K. (2012). Thermal Properties of Graphene : Fundamentals and Applications. MRS Bull..

[cit16] Marsden A. J., Papageorgiou D. G., Vallés C., Liscio A., Palermo V., Bissett M. A., Young R. J., Kinloch I. A. (2018). Electrical Percolation in Graphene–Polymer Composites. 2D Mater..

[cit17] An X., Simmons T., Shah R., Wolfe C., Lewis K. M., Washington M., Nayak S. K., Talapatra S., Kar S. (2010). Stable Aqueous Dispersions of Noncovalently Functionalized Graphene from Graphite and Their Multifunctional High-Performance Applications. Nano Lett..

[cit18] Woltornist S. J., Adamson D. H. (2016). Properties of Pristine Graphene Composites Arising from the Mechanism of Graphene-Stabilized Emulsion Formation. Ind. Eng. Chem. Res..

[cit19] Bento J. L., Brown E., Woltornist S. J., Adamson D. H. (2017). Thermal and Electrical Properties of Nanocomposites Based on Self-Assembled Pristine Graphene. Adv. Funct. Mater..

[cit20] Cui X., Ding P., Zhuang N., Shi L., Song N., Tang S. (2015). Thermal Conductive and Mechanical Properties of Polymeric Composites Based on Solution-Exfoliated Boron Nitride and Graphene Nanosheets: A Morphology-Promoted Synergistic Effect. ACS Appl. Mater. Interfaces.

[cit21] Kargar F., Barani Z., Salgado R., Debnath B., Lewis J. S., Aytan E., Lake R. K., Balandin A. A. (2018). Thermal Percolation Threshold and Thermal Properties of Composites with High Loading of Graphene and Boron Nitride Fillers. ACS Appl. Mater. Interfaces.

[cit22] Maleki M., Shokouhimehr M., Karimian H., Beitollahi A. (2016). Three-Dimensionally Interconnected Porous Boron Nitride Foam Derived from Polymeric Foams. RSC Adv..

[cit23] Vishakh M. G., Painuly D., Ragupathy L., Kanappally A., Vadivelu P. (2025). Boron Nitride and Few Layers Graphene Incorporated Carboxylated Butadiene Nitrile Rubber Hybrid Nanocomposites: Preparation, Properties and Mechanistic Insights. Polym. Compos..

[cit24] Nallamilli T., Binks B. P., Mani E., Basavaraj M. G. (2015). Stabilization of Pickering Emulsions with Oppositely Charged Latex Particles: Influence of Various Parameters and Particle Arrangement around Droplets. Langmuir.

[cit25] Nallamilli T., Mani E., Basavaraj M. G. (2014). A Model for the Prediction of Droplet Size in Pickering Emulsions Stabilized by Oppositely Charged Particles. Langmuir.

[cit26] Beerbower A. (1971). Surface Free Energy: A New Relationship to Bulk Energies. J. Colloid Interface Sci..

[cit27] Chen C., Cai W., Long M., Zhou B., Wu Y., Wu D., Feng Y. (2010). Synthesis of Visible-Light Responsive Graphene Oxide/TiO_2_ Composites with p/n Heterojunction. ACS Nano.

[cit28] Tang M., Wu T., Xu X., Zhang L., Wu F. (2014). Factors That Affect the Stability, Type and Morphology of Pickering Emulsion Stabilized by Silver Nanoparticles/Graphene Oxide Nanocomposites. Mater. Res. Bull..

[cit29] Coleman J. N., Lotya M., O'Neill A., Bergin S. D., King P. J., Khan U., Young K., Gaucher A., De S., Smith R. J., Shvets I. V., Arora S. K., Stanton G., Kim H.-Y., Lee K., Kim G. T., Duesberg G. S., Hallam T., Boland J. J., Wang J. J., Donegan J. F., Grunlan J. C., Moriarty G., Shmeliov A., Nicholls R. J., Perkins J. M., Grieveson E. M., Theuwissen K., McComb D. W., Nellist P. D., Nicolosi V. (2011). Two-Dimensional Nanosheets Produced by Liquid Exfoliation of Layered Materials. Science.

[cit30] Fan Y., Cho U. R. (2019). Effects of Graphite and Boron Nitride Based Fillers on Mechanical, Thermal Conductive, and Thermo-physical Properties in Solution Styrene–Butadiene Rubber. Polym. Compos..

[cit31] Shao L., Shi L., Li X., Song N., Ding P. (2016). Synergistic Effect of BN and Graphene Nanosheets in 3D Framework on the Enhancement of Thermal Conductive Properties of Polymeric Composites. Compos. Sci. Technol..

[cit32] An F., Li X., Min P., Li H., Dai Z., Yu Z.-Z. (2018). Highly Anisotropic Graphene/Boron Nitride Hybrid Aerogels with Long-Range Ordered Architecture and Moderate Density for Highly Thermally Conductive Composites. Carbon.

[cit33] Denkov N. D., Ivanov I. B., Kralchevsky P. A., Wasan D. T. (1992). A Possible Mechanism of Stabilization of Emulsions by Solid Particles. J. Colloid Interface Sci..

[cit34] Hui T., Adamson D. H. (2020). Kinetic Study of Surfactant-Free Graphene Exfoliation at a Solvent Interface. Carbon.

[cit35] Woltornist S. J., Oyer A. J., Carrillo J. M. Y., Dobrynin A. V., Adamson D. H. (2013). Conductive Thin Films of Pristine Graphene by Solvent Interface Trapping. ACS Nano.

[cit36] Brown E. E. B., Woltornist S. J., Adamson D. H. (2020). PolyHIPE Foams from Pristine Graphene: Strong, Porous, and Electrically Conductive Materials Templated by a 2D Surfactant. J. Colloid Interface Sci..

[cit37] Cameron N. R. (2005). High Internal Phase Emulsion Templating as a Route to Well-Defined Porous Polymers. Polymer.

[cit38] Menner A., Bismarck A. (2006). New Evidence for the Mechanism of the Pore Formation in Polymerising High Internal Phase Emulsions or Why polyHIPEs Have an Interconnected Pore Network Structure. Macromol. Symp..

[cit39] Feng W., Chai Y., Forth J., Ashby P. D., Russell T. P., Helms B. A. (2019). Harnessing Liquid-in-Liquid Printing and Micropatterned Substrates to Fabricate 3-Dimensional All-Liquid Fluidic Devices. Nat. Commun..

[cit40] Li Y., Liu X., Zhang Z., Zhao S., Tian G., Zheng J., Wang D., Shi S., Russell T. P. (2018). Adaptive Structured Pickering Emulsions and Porous Materials Based on Cellulose Nanocrystal Surfactants. Angew. Chem., Int. Ed..

