# Supplementary material for: Hierarchical assembly of mixed 2D surfactants in polyHIPEs: tuning conductive networks through multi-scale structuring
Source: RSC Adv. 2026 Apr 28;16(24):22216–26. doi: 10.1039/d6ra01377e (PMC13123643; doi:10.1039/d6ra01377e)
Supplement: RA-016-D6RA01377E-s001 [file RA-016-D6RA01377E-s001.pdf]

Supplementary Information (SI) for RSC Advances.
This journal is © The Royal Society of Chemistry 2026

Supporting information for:

Hierarchical Assembly of Mixed 2D Surfactants in PolyHIPEs: Tuning Conductive Networks through Multi-scale Structuring

Deep Shikha Srivastava†, Elizabeth E. B. Brown‡, Deepthi Varghese†, Douglas H. Adamson†,‡,*

† Department of Chemistry, University of Connecticut, Storrs, CT 06269, USA
‡ Polymer Program, Institute of Materials Science, University of Connecticut, Storrs, CT 06269

Composite	1 μm graphite (g)	10 μm graphite (g)	DI water (mL)	styrene (mL)	DVB (mL)	AIBN (g)
100% 1 μm	0.88	0	140	60	14	0.18
75% 1 μm	0.66	0.22	140	60	14	0.18
50% 1 μm	0.44	0.44	140	60	14	0.18
25% 1 μm	0.22	0.66	140	60	14	0.18
100% 10 μm	0	0.88	140	60	14	0.18

Table S1: Reagent amounts for “pre-mixed” graphite composites. “Pre-mixed” refers to composites in which both the 1 and 10 μm graphites were combined in the same Erlenmeyer flask prior to forming an emulsion.

Composite	1 μm graphite (g)	DI water (mL)	styrene (mL)	DVB (mL)	AIBN (g)
100% 1 μm	0.88	140	60	14	0.18
75% 1 μm	0.66	105	45	10.5	0.135
50% 1 μm	0.44	70	30	7.0	0.09
25% 1 μm	0.22	35	15	3.5	0.045
100% 10 μm	0.00	0	0	0.0	0.00

Table S2: Reagent amounts for 1 μm emulsions used in the “post-mixed” graphite composites. “Post-mixed” refers to emulsions of each graphite size (1 and 10 μm) being made separately, then combined once an emulsion was formed.

Composite	10 µm graphite (g)	DI water (mL)	styrene (mL)	DVB (mL)	AIBN (g)
**100% 1 µm**	0.00	0	0	0.0	0.00
**75% 1 µm**	0.22	35	15	3.5	0.045
**50% 1 µm**	0.44	70	30	7.0	0.09
**25% 1 µm**	0.66	105	45	10.5	0.135
**100% 10 µm**	0.88	140	60	14.0	0.18

Table S3: Reagent amounts for 10 µm emulsions used in the "post-mixed" graphite composites. "Post-mixed" refers to emulsions of each graphite size (1 and 10 µm) being made separately, then combined once an emulsion was formed.

Composite	2D surfactant (g)	DI water (mL)	styrene (mL)	DVB (mL)	AIBN (g)
**Graphite**	0.44	70	30	7.0	0.09
**Boron nitride**	0.88	70	30	7.0	0.09
**Pre-mixed BNG**	0.22 G; 0.22 BN	70	30	7.0	0.09
**Post-mixed: G**	0.22	35	15	3.5	0.045
**Post-mixed: BN**	0.44	35	15	3.5	0.045

Table S4: Reagent amounts for graphite and boron nitride emulsions, including pre- and post-mixed samples.

Composite	Pre-mixed average electrical conductivity (S/m)	Post-mixed average electrical conductivity (S/m)
**100% 1 µm**	0.135 ± 0.005	0.135 ± 0.005
**75% 1 µm**	0.124 ± 0.004	0.129 ± 0.001
**50% 1 µm**	0.194 ± 0.008	0.140 ± 0.006
**25% 1 µm**	0.244 ± 0.029	0.170 ± 0.003
**100% 10 µm**	0.309 ± 0.013	0.309 ± 0.013

Table S5: Electrical conductivity values for 1 and 10 µm graphene composites with varying percentages of each type of graphene, as an accompaniment to Figure 4 in the main manuscript.

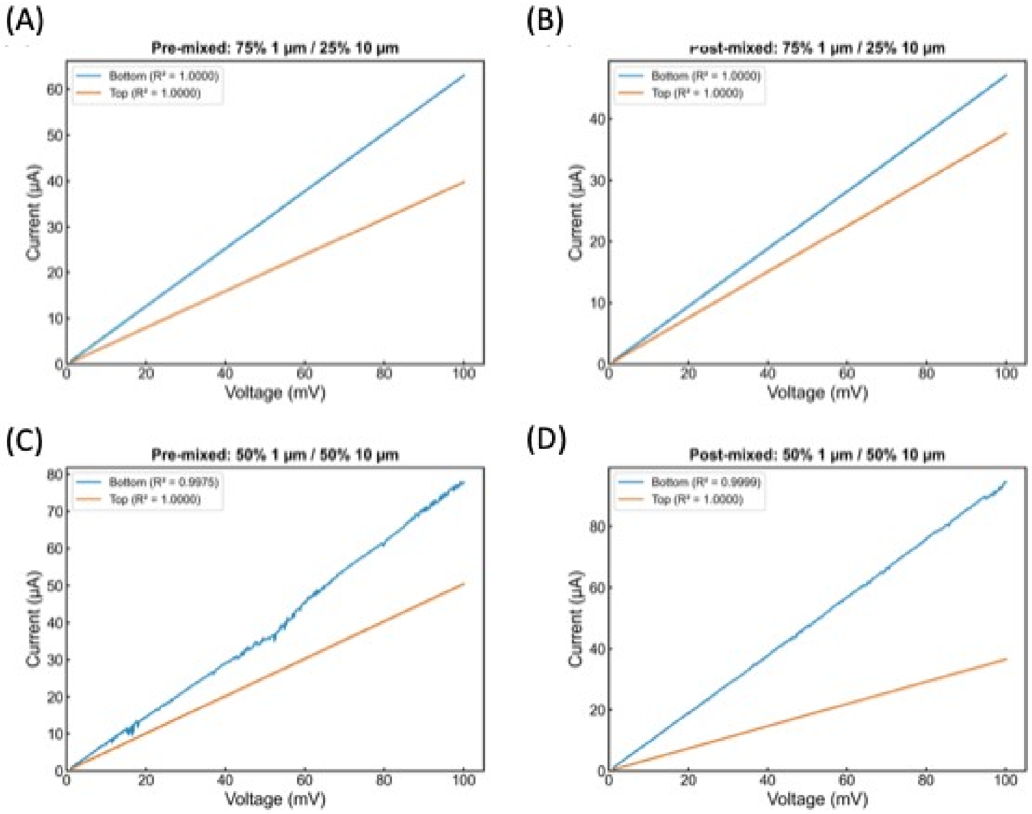

Figure SI1. Representative IV curves of samples reported in Figure 4b.

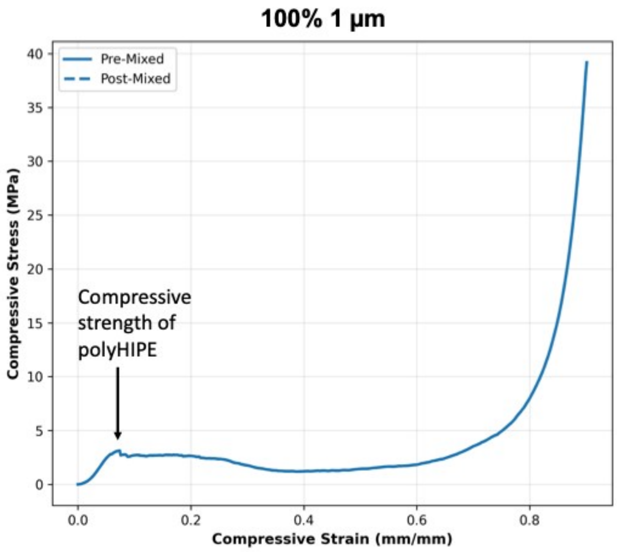

Figure SI2. Example stress/strain curve used to determine the compressive strength of the graphene/BN composites.

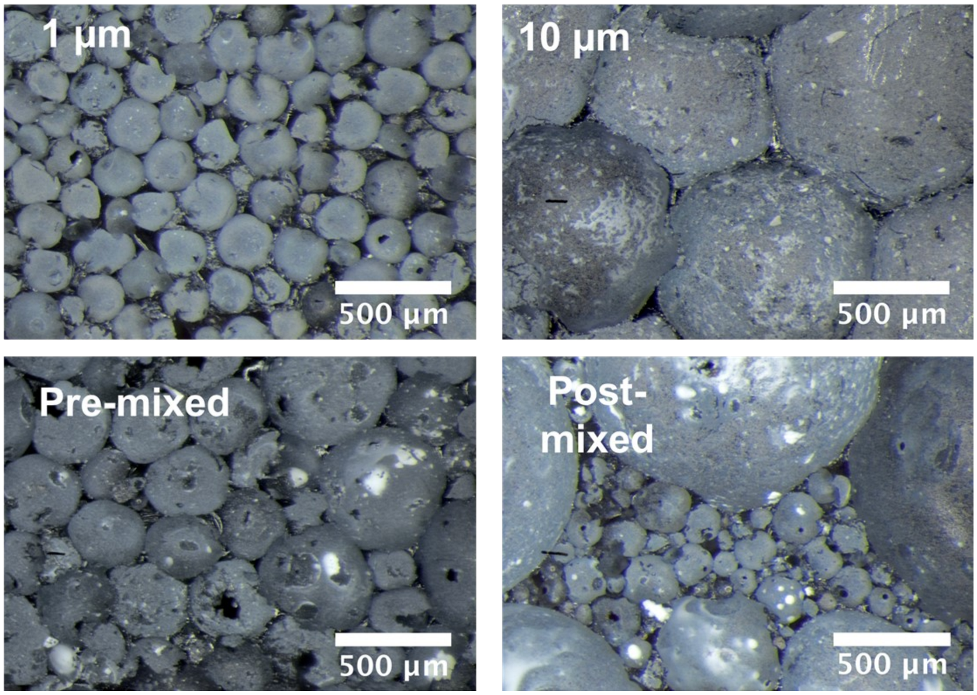

Figure SI3: An enlarged view of the photos of 1 µm, 10 µm, pre-mixed, and post-mixed graphite composites shown in Figure 2 of the manuscript, presented for easier viewing.

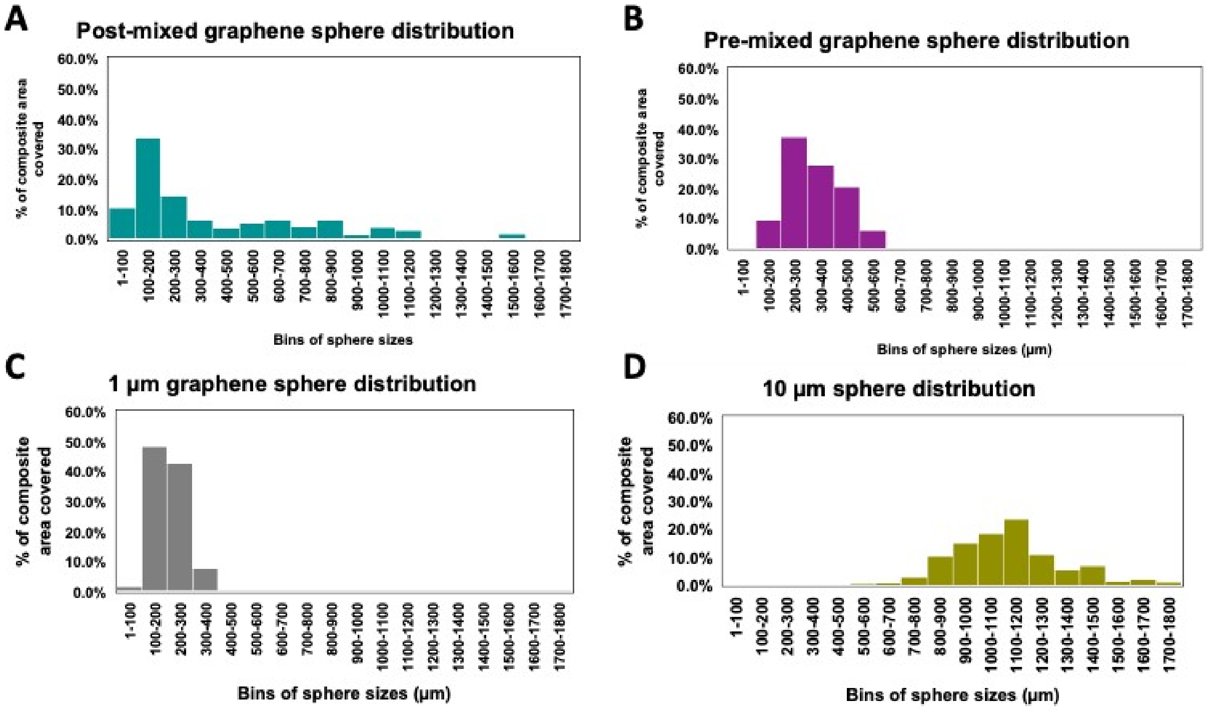

Figure SI4. Histograms of sphere size distributions for A) polyHIPE made by mixing emulsions made separately with 1 µm and 10 µm graphite flakes, B) polyHIPE made by mixing 1 µm and 10 µm graphite flake prior to forming an emulsion, C) polyHIPE formed from 1 µm graphite flake, and D) polyHIPE made with 10 µm graphite flakes. The vertical axis is the % area of the images constituting each bin of sphere sizes.

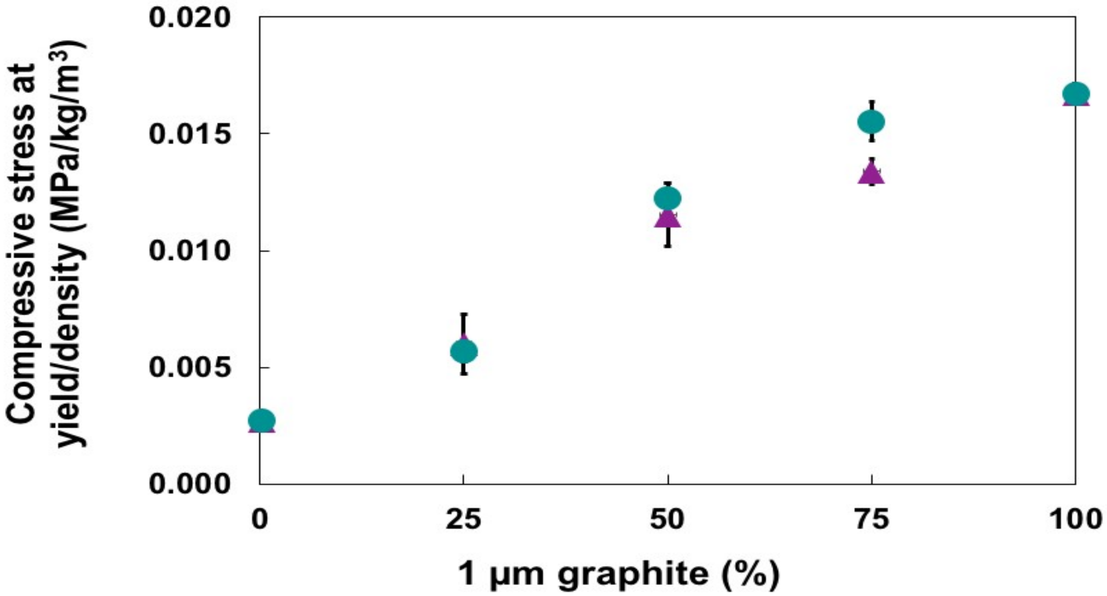

Figure SI5. Graph depicting the change in specific compressive stress (as normalized by composite density) of composites made with varying fractions of 1 µm graphite. The pre-mixed samples are represented by purple triangles, and the post-mixed materials correspond to the teal circles.

Composite (% 1 µm graphite)	Post-mixed density (g/cm^3)	Pre-mixed density (g/cm^3)	Porosity range (%)
100	0.196	0.196	81
75	0.176	0.161	83–85
50	0.140	0.150	86–87
25	0.089	0.103	90–92
0 (100% 10µ Graphite)	0.077	0.077	93

Table S6: Density and porosity of samples whose compressive strength and conductivity are plotted in Figure 4.

Density was calculated by dividing the sample mass by its volume, measured with calipers. Porosity was found using the standard foam porosity formula:

$$\textbf{Porosity} = 1 - \left(\frac{\rho_{foam}}{\rho_{solid}}\right)$$

Where:

- $\boldsymbol{\rho_{foam}}$ = measured density of the composite foam (from the data, e.g. 0.196 g/cm^3)
- $\boldsymbol{\rho_{solid}}$ = density of the fully dense matrix material

It was assumed that $\rho_{solid} \approx 1.05\ g/cm^3$ for crosslinked polystyrene-divinylbenzene, which is a standard literature value for PS-DVB.

For example, for the 100% 1 μm composite (bottom):

Porosity = 1 − $\left(\frac{0.196}{1.05}\right)$ = 1 − 0.187 = 0.813 → **~81%**
